# *Mashua* (*Tropaeolum tuberosum* Ruiz & Pavón): Nutritional Composition, Bioactive Compounds, and Functional Potential as an Andean Natural Ingredient

**DOI:** 10.3390/foods14244198

**Published:** 2025-12-06

**Authors:** Suny Luera-Quiñones, María Jimena Correa, Cesar Moreno-Rojo, Rebeca Salvador-Reyes, Luz María Paucar-Menacho

**Affiliations:** 1 Programa de Doctorado en Ingeniería Agroindustrial Mención Transformación Avanzada de Granos y Tubérculos Andinos, Universidad Nacional del Santa, Chimbote 02712, Peru; 2024818018@uns.edu.pe (S.L.-Q.); rsalvador@uns.edu.pe (R.S.-R.); 2 Centro de Investigación y Desarrollo en Ciencia y Tecnología de los Alimentos (CIDCA), Facultad de Ciencias Exactas-Universidad Nacional de La Plata, Comisión de Investigaciones Científicas de la Provincia de Buenos Aires (CIC), Consejo Nacional de Investigaciones Científicas y Técnicas (CONICET), 47 y 116, La Plata CP 1900, Argentina; mjcorrea@biol.unlp.edu.ar; 3 Departamento Académico de Agroindustria y Agronomía, Facultad de Ingeniería, Universidad Nacional del Santa, Chimbote 02712, Peru; cmoreno@uns.edu.pe

**Keywords:** Andean tuber, anthocyanins, añu, cubio, functional foods, food safety, glucosinolates, isaño, underutilized and neglected crops

## Abstract

*Mashua* (*Tropaeolum tuberosum*), an underutilized Andean tuber, has gained increasing scientific interest due to its exceptional nutritional composition and high bioactive phytochemical content. This narrative review synthesizes evidence from 68 peer-reviewed studies published up to August 2025, obtained through searches in Scopus and Web of Science, examining its macro- and micronutrient profile, richness in starch, proteins, and vitamin C, and diverse bioactive compounds, including glucosinolates, anthocyanins, and flavonoids. These metabolites exhibit strong antioxidant, anti-inflammatory, anticancer, and cardioprotective activities, with the highest concentrations being observed in purple and black morphotypes. Recent studies demonstrated that incorporating *mashua* into bakery, extruded, and fermented food formulations enhanced nutritional value and oxidative stability. Overall, *mashua* represents a promising natural ingredient for functional foods and nutritional applications. Further research is required to optimize processing conditions, improve the stability and bioavailability of its active compounds, and validate its health-promoting effects.

## 1. Introduction

*Mashua* (*Tropaeolum tuberosum* Ruiz & Pavón), also known as *añu*, *isaño*, or *cubio*, is an Andean tuber that has been cultivated in Peru, Bolivia, Ecuador, and Colombia since pre-Hispanic times. This traditional crop has been valued for its remarkable adaptability to the agroecological conditions of the high Andes and for its nutritional and medicinal properties, becoming an essential component of both the diet and cultural identity of Andean communities [1]. According to archeological evidence discovered in the Huachumachay cave, located in the Jauja Valley of Peru, the domestication of *mashua* dates back more than 7500 years [2]. It is cultivated at altitudes ranging from 2800 to 4100 m above sea level and thrives in cold climates and nutrient-poor soils [3]. Although primarily grown in Peru, Bolivia, Ecuador, and Colombia, its presence in Argentina and Chile has also been reported in smaller quantities [1].

*Mashua* is recognized for its high nutritional value. It contains a significant proportion of carbohydrates, mainly starch, representing approximately 78.6% of its dry weight. It also has a considerable protein content, ranging from 11.4 to 16 g per 100 g dry weight [4]. Regarding micronutrients, it has a particularly high vitamin C content, reaching up to 476 mg per 100 g fresh weight, higher than that of other tubers such as potato (*Solanum tuberosum*) [5]. Furthermore, it is a source of minerals such as calcium (35–52 mg/100 g DW), phosphorus (115–180 mg/100 g), and iron (7–8 mg/100 g), which are essential for various physiological functions [4].

*Mashua* contains bioactive compounds that confer antioxidant and medicinal properties beyond its nutritional profile. Glucosinolates and isothiocyanates are known for their antioxidant activity and potential anticancer effects [6]. The black variety of *mashua*, in particular, exhibits a high content of anthocyanins, which are natural pigments with strong antioxidant activity, making it especially beneficial for cardiovascular health and preventing oxidative stress-related diseases [7].

Traditionally, *mashua* has been used in Andean folk medicine to treat various ailments. It is attributed with diuretic, anti-inflammatory, and antimicrobial properties and has been used for the treatment of urinary tract infections and kidney disorders [7]. However, recent studies have reported its possible adverse effects on male reproductive function in animal models. In rats fed with the tuber or hydroalcoholic extracts of *mashua*, a significant reduction in testosterone levels, sperm production, and sperm motility was observed, which is a phenomenon associated with the presence of isothiocyanates (benzyl-type glucosinolates) [6].

*Mashua* represents a valuable opportunity for dietary diversification and functional product development in the food industry. Its high content of antioxidants and bioactive compounds makes it a promising ingredient for the formulation of foods with health-promoting properties. Likewise, the pharmaceutical industry could benefit from further research and development of products based on the bioactive compounds of *mashua,* particularly in the prevention and management of chronic diseases [6].

However, current research on *mashua* remains dispersed and focuses on isolated aspects such as composition, specific phytochemicals, or particular processing methods. There is no integrated analysis that compares morphotypes, evaluates the impact of processing on their functional properties, or synthesizes their potential applications as a functional ingredient. This lack of unified evidence limits its role in technological valorization and broader use in the food industry. Therefore, this review aims to compile and analyze the nutritional composition, bioactive compounds, antioxidant capacity, and potential food applications of *mashua*, emphasizing its potential as a functional food and its contribution to human health.

## 2. Methodology

This work was developed as a narrative review aimed at synthesizing and critically interpreting scientific evidence related to the nutritional composition, bioactive compounds, functional properties, and potential food applications of *T. tuberosum*. Although it does not follow the structure of a systematic review, the study adheres to methodological transparency standards that ensure reproducibility and scholarly rigor. The literature search was conducted primarily in the Scopus and Web of Science Core Collection databases, which were selected due to their broad coverage of peer-reviewed scientific publications. Additional verification of reference lists and citation chains was performed through Google Scholar to identify complementary studies not indexed in subscription databases.

The search strategy relied on combinations of the species’ scientific and common names, together with terms associated with nutritional, functional, and phytochemical characteristics. Queries such as “Tropaeolum tuberosum” AND (nutritional composition OR bioactive compounds OR phenolics OR glucosinolates OR antioxidants) and (*mashua* OR *isaño* OR *añu* OR *cubio*) AND (starch OR protein OR vitamins OR minerals OR glucosinolates OR anthocyanins) were applied without temporal restrictions, allowing the inclusion of both foundational and contemporary studies. Searches were conducted for studies up to August 2025. All retrieved publications were exported to a reference manager for screening.

The selection of studies followed predefined inclusion criteria focused on species’ specificity, analytical rigor, and relevance to this review’s nutritional and functional scope. Articles were included when they provided quantitative or qualitative data on proximate composition, bioactive compound profiling, antioxidant capacity, and techno-functional behavior of *mashua* or its derivatives, or when they addressed traditional uses and food applications supported by analytical or experimental evidence. Publications available in English or Spanish were considered eligible. The exclusion criteria were as follows: reviews, articles without experimental data, studies focused solely on agronomic or clinical aspects without a connection to food applications, and documents with incomplete information or unverifiable results.

The screening process followed a Preferred Reporting Items for Systematic Reviews-adapted workflow suitable for narrative reviews. An initial 132 records were identified in the databases. After removing duplicates, 104 unique studies were screened by title and abstract, resulting in 87 records being retained for full-text assessment. Of these, 19 were excluded due to insufficient methodological detail, lack of species’ specificity, or limited analytical relevance. Ultimately, 68 scientific articles met all eligibility criteria and were included in the review.

Data extraction was performed manually and organized into thematic matrices covering proximate composition, protein, carbohydrate, lipid, vitamin, and mineral content, phenolic compounds, flavonoids, anthocyanins, and glucosinolate types and concentrations, antioxidant capacity measured by various chemical assays, and results from studies evaluating technological performance and food applications. The synthesis was conducted qualitatively, emphasizing observed trends across genotypes, ecotypes, processing conditions, and agroecological origins rather than applying statistical aggregation, due to significant variability in analytical methods, units, and expression bases (fresh versus dry weight). This methodological structure ensures coherence, transparency, and scientific consistency throughout the review.

## 3. Data Harmonization and Analysis of Variability

The compositional and phytochemical data compiled in this review were derived from studies that used heterogeneous analytical procedures, reporting formats, and expression units. Differences related to methodological variability were carefully examined to ensure coherence in the synthesis and accurate interpretation of results. Across the literature, proximate composition parameters such as protein, lipids, fiber, and ash are typically determined following AOAC-approved protocols, yet variations in laboratory procedures, moisture determination methods, or sample pre-processing (e.g., drying temperature, particle size) influence final values. A broader range of techniques is reported for non-nutrient constituents, including phenolic compounds, flavonoids, anthocyanins, carotenoids, and glucosinolates, including spectrophotometric assays, HPLC-DAD, HPLC-MS/MS, and gravimetric or enzymatic determinations, each affecting quantification sensitivity and comparability.

To address inconsistencies in reporting units, values expressed on a fresh weight (FW) basis were kept as reported by the original studies, whereas data reported on a dry weight (DW) basis were similarly preserved. Given the substantial moisture variability among *mashua* accessions, no conversions between FW and DW were performed unless explicitly indicated or calculable based on the original authors’ data. As such, all tables clearly state the basis and units used to avoid misinterpretation and maintain fidelity to the primary sources.

Variability arising from extraction solvents and analytical methods was also considered. Studies evaluating phenolic and glucosinolate contents have employed solvents such as methanol, ethanol, acetone, aqueous mixtures, or buffered solutions, all of which differ in polarity and extraction efficiency. Additionally, the quantification of phenolics relies on calibration standards such as gallic acid, catechin, or cyanidin-3-glucoside equivalents, while glucosinolates may be expressed as sinigrin or benzyl glucosinolate equivalents. To avoid erroneous cross-study comparisons, values were compiled without recalculating equivalent transformations unless provided in the source.

Finally, methodological differences were explicitly noted when incorporating data into tables and narrative synthesis to contextualize variability across genotypes, ecotypes, and geographic origins. Studies employing incompatible or noncomparable analytical techniques were not merged, and where appropriate, variability is discussed qualitatively rather than quantitatively. This harmonization strategy ensures transparency and supports robust interpretation despite inherent heterogeneity in analytical methodologies.

## 4. Andean Tubers and Roots

**Figure 1:**
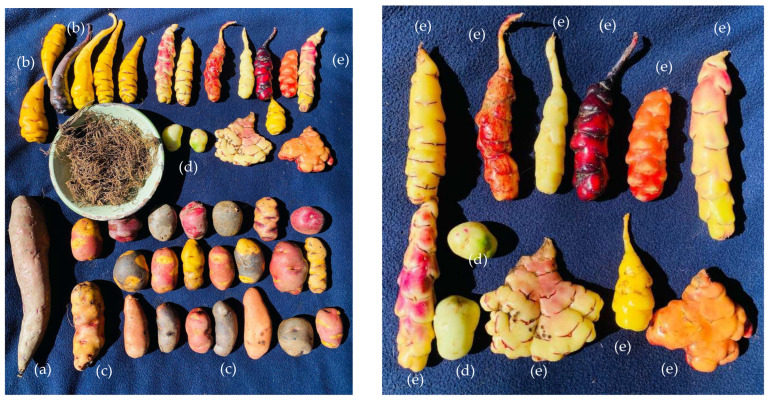
Andean tuber and root varieties: (**a**) *yacón*, (**b**) *mashua*, (**c**) sweet potato, (**d**) *melloco*, and (**e**) *oca* collected from the *Huancaspata* district, *Pataz* province, *La Libertad* region, Peru.

Andean tubers and roots (Figure 1), such as cassava (*Manihot esculenta*), sweet potato (*Solanum tuberosum*), *arracacha* (*Arracacia xanthorrhiza*), *oca* (*Oxalis tuberosa*), *mashua* (*Tropaeolum tuberosum*), *melloco* (*Ullucus tuberosus*), and *yacón* (*Smallanthus sonchifolius*), are essential sources of starch, dietary fiber, and bioactive compounds with diverse functional and nutritional applications. Several studies have demonstrated that these native crops possess prebiotic, antioxidant, and energy-providing properties, making them valuable resources for both human nutrition and the food industry. For example, *yacon* is particularly rich in fructooligosaccharides (FOS), which support intestinal microbiota balance, while *mashua* stands out for its high antioxidant capacity. Starches from these tubers can replace chemically modified variants, offering a more natural and health-oriented alternative. However, environmental factors, such as drought, can negatively affect the growth and quality of staple crops, such as potatos. The broad genetic variability within these tubers enables certain species to better withstand adverse conditions, underscoring their relevance for food security in the Andean region [8].

Despite their remarkable nutritional and cultural value, many of these tubers remain underutilized, and their commercialization remains limited. Recently, South American gastronomy has begun to revalue these native crops, with chefs incorporating them into innovative culinary preparations that highlight their versatility and distinctive flavors. However, limited market availability and low consumer awareness continue to hinder their widespread adoption. Promoting their consumption requires strengthening production systems, improving commercialization chains, and enhancing consumer education about their nutritional and functional benefits. These crops not only offer an opportunity to improve dietary quality but also play a key role in preserving Andean agricultural traditions and fostering sustainable production systems [9,10].

## 5. Tropaeolum Tuberosum

### 5.1. Morphology and Taxonomy

**Figure 2:**
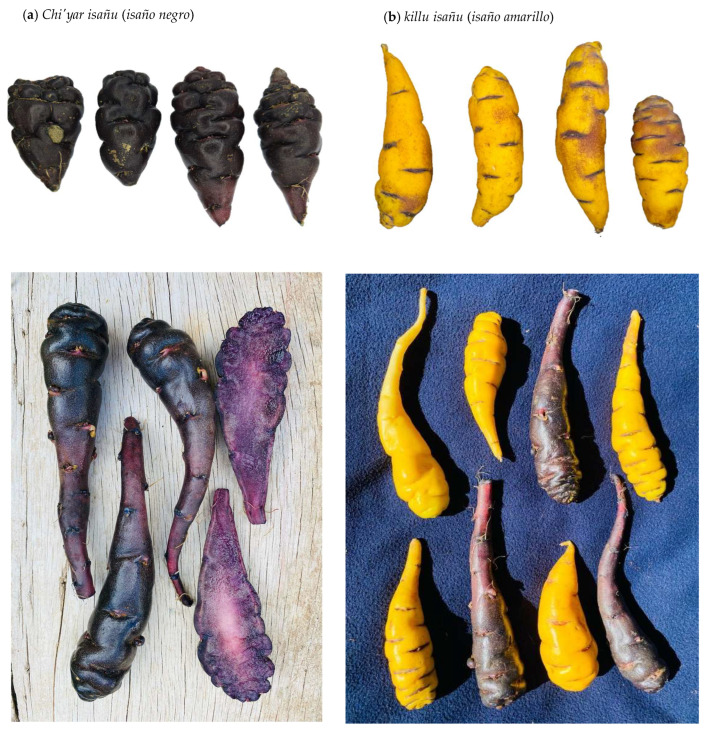
*Mashua* varieties: (**a**) *Chi’yar isaño* (black mashua) and (**b**) *Killu isaño* (yellow mashua) collected from the *Huancaspata* district, *Pataz* province, *La Libertad* region, Peru.

*Mashua* is a perennial herbaceous plant with twining stems and peltate leaves. It is characterized by high phenotypic diversity in the shape, color, and size of its tubers [11]. Several color variants, including yellow, white, purple, and intermediate bicolored forms, have been identified (Figure 2), suggesting a wide genetic base within the species [11]. The morphology of *mashua* tubers varies from elongated and cylindrical to oval or compact. Their surface may be smooth or slightly rough, with shallow eyes distributed along the tuber [12].

The leaves are alternate, entire-margined, and orbicular-peltate, with long, pubescent petioles. *Tropaeolum tuberosum* belongs to the family Tropaeolaceae and is related to other species of the same genus, such as *Tropaeolum majus* and *Tropaeolum pentaphyllum*. It is taxonomically distinguished by its tuber morphology and specialized floral structure, which facilitates differentiation from other Andean tubers such as *oca* (*Oxalis tuberosa*) and *melloco* (*Ullucus tuberosus*) [13].

The morphological and taxonomic characteristics of *T. tuberosum* are summarized in Table 1.

### 5.2. Nutritional Composition

The proximate composition of *mashua*, expressed on a dry weight basis (Table 2), is characterized by a predominance of carbohydrates (≈69.7–79.5 g/100 g DW), followed by protein (≈6.9–15.7 g/100 g DW), crude fiber (≈7.8–8.6 g/100 g DW), ash(≈ 4.0–6.5 g/100 g DW), and low total lipid content (≈0.1–1.4 g/100 g DW) [6]. The starch fraction contains approximately 27% amylose (in isolated starch) and has a relatively small granule size, which determines its functional properties, such as water absorption capacity, swelling power, and solubility. These parameters influence the texture, gelatinization behavior, and rate of intraluminal enzymatic hydrolysis [20].

Despite variability among Andean tubers, the water solubility and water absorption indices of *mashua* starch have been shown to increase progressively at temperatures above ~50 °C. This behavior reflects the disruption of hydrogen bonds and the exposure of hydroxyl groups, enhancing their interaction with the solvent, a process that explains the rheological transition during cooking and its impact on the digestibility of gelatinized starch [20].

From a nutritional and functional standpoint, the combination of a predominantly digestible starch fraction (≈85%) with a resistant fraction (≈15%), a naturally low lipid content, and proteins of moderate biological quality suggests that when consumed as part of mixed dishes, *mashua* provides rapidly available energy and fermentable fiber beneficial for gut microbiota. Its moderate protein fraction can nutritionally complement SAA-rich sources such as cereals or legumes. In addition, technological processes (drying, milling, extrusion) are known to modulate the water and fat absorption capacities of the matrix, affecting the structure and digestion kinetics [6,21,22].

**Table 2:** Proximate composition of *T. tuberosum* by ecotype and geographical origin.

Accession/Ecotype	Origin/Location	Sample Type	Carbohydrates (g/100 g)	Proteins (g/100 g)	Fats (g/100 g)	Fiber (g/100 g)	Ash (g/100 g)	Moisture (%)	Ref.
INIAP-ECU-*izaño*	Ecuador	Tuber DW	78.0 ± 0.5	12.0 ± 0.4	1.1 ± 0.2	6.8 ± 0.1 (TDF)	3.7	86%	[10]
Purple	Collao, Peru	Tuber DW	70.73 ± 0.16	11.72 ± 0.05	4.53 ± 0.06	6.36 ± 0.03	6.66 ± 0.08	89.72 ± 0.62	[4]
Purple	Chucuito, Peru	Tuber DW	76.99 ± 0.21	7.41 ± 0.01	4.40 ± 0.13	5.89 ± 0.02	5.32 ± 0.05	74.51 ± 0.07	
Purple	Puno, Peru	Tuber DW	75.96 ± 0.30	7.86 ± 0.01	4.57 ± 0.02	5.78 ± 0.14	5.84 ± 0.13	84.83 ± 0.47	
Purple	Yunguyo, Peru	Tuber DW	74.96 ± 0.02	8.16 ± 0.06	4.70 ± 0.01	5.79 ± 0.10	6.40 ± 0.03	86.72 ± 0.75	
Yellow	Collao, Peru	Tuber DW	74.40 ± 0.11	7.83 ± 0.03	4.60 ± 0.12	5.99 ± 0.12	7.18 ± 0.08	82.86 ± 0.20	
Yellow	Chucuito, Peru	Tuber DW	75.64 ± 0.31	7.58 ± 0.08	5.63 ± 0.02	5.93 ± 0.03	5.22 ± 0.35	86.33 ± 0.28	
Yellow	Puno, Peru	Tuber DW	76.82 ± 0.25	6.96 ± 0.09	4.70 ± 0.05	5.93 ± 0.04	5.60 ± 0.07	86.90 ± 0.71	
Yellow	Yunguyo, Peru	Tuber DW	73.79 ± 0.07	9.98 ± 0.02	4.79 ± 0.01	6.20 ± 0.06	5.23 ± 0.05	87.81 ± 0.21	
Yellow–Purple	Collao, Peru	Tuber DW	77.33 ± 0.03	7.54 ± 0.03	4.57 ± 0.06	5.23 ± 0.03	5.23 ± 0.09	76.11 ± 0.42	
Yellow–Purple	Chucuito, Peru	Tuber DW	77.50 ± 0.17	7.15 ± 0.04	4.62 ± 0.01	5.91 ± 0.06	4.81 ± 0.09	80.85 ± 0.49	
Yellow–Purple	Puno, Peru	Tuber DW	76.98 ± 0.20	7.31 ± 0.09	4.57 ± 0.07	5.85 ± 0.04	5.29 ± 0.01	81.00 ± 0.35	
Yellow–Purple	Yunguyo, Peru	Tuber DW	77.43 ± 0.06	7.95 ± 0.04	4.71 ± 0.04	5.79 ± 0.13	4.12 ± 0.02	83.48 ± 0.21	
Wild Purple *isaño*	Peru (wild)	Tuber DW	76.31 ± 0.07	9.15 ± 0.02	0.82 ± 0.01	6.42 ± 0.02	6.05 ± 0.05	n.d.	[23]
Wild Yellow *isaño*	Peru (wild)	Tuber DW	80.85 ± 1.15	7.14 ± 0.02	0.42 ± 0.01	5.53 ± 0.15	5.08 ± 0.04	n.d.	
Wild Pink *isaño*	Peru (wild)	Tuber DW	79.71 ± 0.01	7.58 ± 0.01	0.31 ± 0.02	6.35 ± 0.04	5.07 ± 0.14	n.d.	
*Zapallo* (cold zone)	Ecuador (3331 m a.s.l.)	Tuber DW	78.35 ± 1.43	9.88 ± 0.72	0.33 ± 0.02	5.26 ± 0.52	n.d.	n.d.	[24]
*Zapallo* (temperate zone)	Ecuador (2865 m a.s.l.)	Tuber DW	81.13 ± 2.25	9.73 ± 0.15	0.42 ± 0.07	4.51 ± 0.39	n.d.	n.d.	
*Zapallo* (warm zone)	Ecuador (2064 m a.s.l.)	Tuber DW	81.38 ± 1.95	7.10 ± 0.72	0.26 ± 0.05	4.51 ± 0.06	n.d.	13.58 ± 1.84	
Poza Rondador Accession	Ecuador	Tuber FW	n.d.	18.25	n.d.	n.d.	n.d.	n.d.	[25]
Milicia Roja Accession	Ecuador	Tuber FW	n.d.	16.25	n.d.	n.d.	n.d.	n.d.	
Green–Yellow Accession	Ecuador	Tuber FW	n.d.	14.13	n.d.	n.d.	n.d.	n.d.	
Purple Accession	Ecuador	Tuber FW	n.d.	12.56	n.d.	n.d.	n.d.	n.d.	
Yellow Accession	Ecuador	Tuber FW	n.d.	11.19	n.d.	n.d.	n.d.	n.d.	
White Accession	Ecuador	Tuber FW	n.d.	10.06	n.d.	n.d.	n.d.	n.d.	
*Santo jonk’ori*	Huarina, Omasuyos	Tuber FW	15.3 (calc.)	2.1 ± 0.7	0.30 ± 0.05	n.d.	0.77 ± 0.05	81.5 ± 2.7	[3]
*Achakani*	Huarina, Omasuyos	Tuber FW	13.7 (calc.)	2.3 ± 0.6	0.25 ± 0.02	n.d.	0.78 ± 0.07	83.0 ± 0.5	
*Ch’iyara*	Huarina, Omasuyos	Tuber FW	17.1 (calc.)	1.5 ± 0.7	0.21 ± 0.03	n.d.	0.89 ± 0.06	80.3 ± 1.4	
Black *mashua*	Huancavelica, Peru	Flour DW	67.81	10.31	1.45	11.22	n.d.	n.d.	[26]
Pulverized black *mashua*	Acobamba, Huancavelica	Dehydrated flour DW	75.19 ± 0.20	9.01 ± 0.12	1.15 ± 0.10	6.54 ± 0.12 (CF)	n.d.	7%	
Yellow Ecotype	Paucará, Huancavelica	Tuber FW	9.97 (by diff.)	0.98 ± 0.03	0.13 ± 0.03	n.d.	n.d.	n.d.	[27]
*Mashua* flour	Ambato, Ecuador	Flour	56.89 ± 0.25	9.12 ± 0.13	0.59 ± 0.07	9.60 ± 0.05	n.d.	n.d.	[14]
*Mashua*	Peru	Tuber DW	69.05 (41.35 starch + 27.70 sugar)	9.21 ± 0.18	0.92 ± 0.04	15.59 (5.04 sol. + 10.55 insol.)	n.d.	n.d.	[20]
*Mashua* purée	Ecuador	Cooked purée FW	36.56 ± 0.02 (NFE)	4.97 ± 0.04	1.08 ± 0.01	2.50 ± 0.01 (CF)	n.d.	n.d.	[28]
Isolation of *mashua* starch	Peru	Starch DW	99.56 ± 0.95 (starch)	traces	0.02 ± 0.00	10.55 ± 0.03 (insol.)	n.d.	n.d.	[20]
Average *Mashua* values	General reference	Edible tuber FW	9.7	1.5	0.1	0.8 (CF)	n.d.	~85%	[19,29]

Note: Values are expressed as mean ± standard deviation when available. Abbreviations: DW = dry weight; FW = fresh weight; TDF = total dietary fiber; IDF = insoluble dietary fiber; SDF = soluble dietary fiber; CF = crude fiber; NFE = nitrogen-free extract; n.d. = not determined; calc. = calculated by the difference. The following AOAC methods were used for the proximate analyses: protein analysis by Kjeldahl (N × 6.25), fat analysis by Soxhlet extraction, carbohydrate analysis by difference or the Dubois method, and fiber analysis by enzymatic–gravimetric (TDF) or acid–base (CF) procedures.

#### 5.2.1. Proteins and Amino Acids

In *mashua*, the protein content on a dry weight (DW) basis shows considerable variability across genetic materials and cultivation conditions, ranging from 6.9 to 15.7 g/100 g DW. Using the average moisture content reported for fresh tubers (≈84.5%), this range corresponds to approximately 1.07–2.43 g/100 g on a fresh weight (FW) basis, with an estimated mean of 1.19 g/100 g FW when taking a reference value of 7.7 g/100 g DW (compiled from classical studies) [6]. This variability is nutritionally relevant because it determines the actual contribution of utilizable nitrogen in high-moisture diets, requiring that reported values be interpreted considering both the expression basis and the water content of the samples analyzed.

*Mashua* contains appreciable amounts of lysine (≈35–69 mg/g protein), threonine (≈22–46 mg/g), valine (≈25–88 mg/g), isoleucine (≈25–44 mg/g), leucine (≈35–56 mg/g), tyrosine (≈13–62 mg/g), and tryptophan (≈5–12 mg/g), with variations attributable to genotype and analytical methodology. Overall, these data suggest that sulfur-containing amino acids (methionine + cystine) tend to represent the first limiting factor, followed by tryptophan and, in some cases, leucine, when compared with FAO/WHO reference protein scoring patterns [6]. From a digestive physiology perspective, this composition integrates into a tuber matrix rich in digestible starch (≈85%) and contains a non-absorbable fraction (~15%) that behaves as resistant starch, potentially modulating colonic fermentation and postprandial glycemic response while attenuating the rate of amino-nitrogen delivery to the enterocyte [6]. The low proportion of indigestible galactosides and fiber in these tubers, combined with the presence of digestible free sugars, explains the high overall carbohydrate digestibility in monogastric organisms [21].

Dried *mashua* flour has a high fat absorption index (≈0.95 g oil/g sample), which is linked to its relatively high protein content (≈9.12%) and hydrophobic interactions between non-polar amino acids and hydrocarbon chains. These interactions may improve flavor retention and palatability of developed foods. However, they could also affect gastric emptying and, consequently, modify the digestion kinetics of starch and proteins within complex food matrices [22]. Given the relative limitation of SAAs and tryptophan, the principle of protein complementarity with legumes or pseudocereals rich in these amino acids remains applicable when *mashua* constitutes a significant dietary component.

#### 5.2.2. Carbohydrates

The starch granules of *mashua* exhibit spheroidal and truncated ellipsoidal shapes, with an average size ranging from 5 to 30 µm. Their heterogeneous distribution influences functional and technological properties, making this starch suitable for diverse food industry applications [6]. *Mashua* starch exhibits higher thermal stability and thickening capacity than other Andean tubers [10]. Its notable fructooligosaccharide content suggests potential prebiotic effects beneficial to intestinal health [30]. In addition, it provides up to 27.7% soluble sugar [19].

Native *mashua* starch has been reported to contain 27.44% amylose (72.56% amylopectin), a composition associated with low gelatinization temperatures and good stability during cooling; these properties favor formulations requiring mild thermal processing and no prolonged cold chain (amylose/amylopectin ratio ≈ 0.38) [20]. Complementary studies characterizing *cubio* starch (a local denomination for the same species) found 31.2% amylose (68.8% amylopectin; amylose/amylopectin ≈ 0.45), with a B-type crystalline pattern, and approximately 94% RS in the measured fraction, indicating low susceptibility of native starch to enzymatic hydrolysis under analytical conditions [30]. This discrepancy relative to the previous pattern can be explained by cultivar differences and, above all, by the sample’s thermomechanical history (native versus gelatinized/cooled), the physical scale (powder vs. film), and the quantification methods used, all of which are recognized factors that modulate enzymatic accessibility [30].

Taken together, the amylose range of 27–31% (amylose/amylopectin ≈ 0.38–0.45) places *mashua* starch within the intermediate-to-high amylose category among Andean tubers, with clear technological and nutritional implications [30]. A higher amylose proportion tends to restrict granule swelling and increase final viscosity and gel firmness due to the promotion of linear chain networks and faster retrogradation. This behavior is reflected in *mashua* and *melloco*, which develop higher paste viscosities than potato (*Solanum tuberosum*). Such behavior, linked to their higher amylose content and larger particle size, translates into excellent performance as thickeners or gelling agents in food matrices and biomaterials [31].

In a study analyzing *mashua* starches and flour after gelatinization, the enzymatic digestion rate approached 80%, whereas RS fractions were very low in the native form (≈0.62%), even lower after cooking (≈0.27%), and only partially recovered upon cooling (≈0.51%). These results suggest that the post-cooking structure and chain-length distribution of amylopectin did not promote the extensive formation of retrograded RS3 under those conditions, despite its amylose content [31].

In summary, *mashua* starches typically present apparent amylose contents of ~27–31% (amylose/amylopectin ≈ 0.38–0.45). This combination confers good swelling and thickening capacity owing to amylopectin, along with amylose-modulated gelation and retrogradation potential. In addition, compared with highly crystalline starch sources, they exhibit film-forming properties with relatively high water vapor permeability (WVP) and more mobile molecular chains. However, effective digestibility is extremely sensitive to structural state (native vs. gelatinized/retrograded), B-type crystallinity, and analytical method, potentially ranging from very low RS values after cooking to extremely high RS content in native starches with high amylose and B-type crystalline patterns [32,33].

#### 5.2.3. Lipids and Fatty Acids

The lipid profile of *mashua* has been scarcely explored and is relatively low compared with other Andean tubers. In addition, there is a broad variability in the informed values among studies, which is attributed to genotypic differences, agroecological zone, and extraction methods (e.g., Soxhlet extraction using solvents of varying polarity). In purple, yellow, and yellow–purple genotypes cultivated at 3750–3900 m a.s.l. in Puno (Peru), crude fat content ranged from approximately 4.40 to 5.63 g/100 g DW, varying by genotype and location [4]. However, in other cases, lipid concentrations ranged from 0.1 to 0.4 on a dry weight basis [7].

Beyond quantity, lipid quality stands out. *Mashua* oil exhibits a clear predominance of polyunsaturated fatty acids (≈70.8%), with linoleic n-6 ≈ 48.7% and α-linolenic n-3 ≈ 22.1%, followed by palmitic ≈ 21.2%, oleic ≈ 4.0%, stearic ≈ 1.5%, and cis-vaccenic ≈ 1.3%. This characteristic is particularly noteworthy in high-Andean communities, where energy intake is mainly derived from carbohydrates and vegetable oil availability is limited, positioning *mashua* as a strategic nutritional complement.

The n-6/n-3 ratio (linoleic/α-linolenic ≈ 2.2) falls within the optimal range (<5) for eicosanoid and cardiometabolic balance and is comparable—or even superior—to that of other plant matrices (e.g., rapeseed 2.49; walnut 4.64) [6]. This profile implies, on the one hand, a high susceptibility to oxidation (a trait typical of PUFAs), and on the other hand, a potentially favorable impact on inflammatory modulation and plasma lipid regulation, particularly when dietary n-3 (ALA) offsets excess n-6 intake, although their dietary impact remains limited due to the tuber’s low absolute fat content [6,22]. The co-presence of lipophilic antioxidants in the tuber may help stabilize the lipid fraction during processing and storage while enhancing postprandial bioavailability [6].

From a digestive perspective, the combination of a very low lipid content and a starch-dominant matrix suggests that starch structure and fiber content influence the gastric emptying rate and glycemic response more than lipids. Nevertheless, the presence of these polyunsaturates in processed products (flours, snacks, and extruded blends) may modestly enhance the lipid quality of formulations.

#### 5.2.4. Vitamins

Regarding water-soluble vitamins and carotenoid content, *mashua* exhibited marked variability according to genotype and environment (Table 3). Classic Ecuadorian data report 77.37 mg/100 g FW of vitamin C, confirming its nutritional relevance in fresh form, particularly considering its antioxidant and collagen-supporting functions [6]. On a dry weight basis, a more recent study (HPLC, highland region) reported values ranging from 0.53 to 4.46 mg/g DW (i.e., 53–446 mg/100 g DW). When adjusted for observed moisture content, this corresponds to approximately 6–114 mg/100 g FW, explaining the discrepancies among reports and underscoring the instability of AS during storage, freezing, and cooking (where significant losses occur) [4]. Recent studies have also identified differences in vitamin content across genotypes and agroecological conditions. For example, the purple genotype exhibited higher vitamin C concentrations and greater antioxidant capacity, whereas the yellow and mixed genotypes showed intermediate values [4].

*Mashua* also contains provitamin A (carotenoids) with an average concentration of 73 µg retinol equivalents/100 g, placing it among the native root and tuber species with the highest carotenoid density [20]. These compounds have antioxidant and protective functions against oxidative stress, with potential implications for the prevention of chronic diseases.

Carotenoid data show high methodological and genetic heterogeneity: on a fresh basis, β-carotene contents around 10 µg/100 g have been reported historically, whereas in dried matrices and flours, total carotenoids (expressed as β-carotene equivalents) can range from ≈1.3 mg/100 g DW to higher values depending on morphotype and processing. In particular, yellow genotypes have been reported to have higher total carotenoid contents, which is consistent with the concentration effect during drying and solid enrichment [6,22,29].

These vitamins and provitamins contribute to the total antioxidant capacity of *mashua* beyond their classic roles as redox cofactors and retinoid precursors (measured over a broad range via ABTS/ORAC). This antioxidant potential has implications for oxidative stability in food systems and possibly for tissue-level protection against oxidative stress, although the magnitude of these effects depends on bioavailability and the food matrix in which the compounds are consumed [6].

#### 5.2.5. Mineral Content

According to the compiled analytical data for *mashua* (Table 3), on a dry weight basis, mineral concentrations are approximately K ≈ 1.99% (≈1990 mg/100 g DW), P ≈ 0.32%, Mg ≈ 0.11%, and Na ≈ 0.044%, along with trace levels of Fe (~42 ppm), Zn (~48 ppm), Cu (~9 ppm), and Mn (~7 ppm). When converted to an FW basis, assuming 84.5% moisture, these values correspond to approximately K ≈ 300 mg/100 g FW, P ≈ 50 mg/100 g FW, and Mg ≈ 17 mg/100 g FW, with proportionally reduced microelements. These figures align with the role of Andean tubers as moderate mineral sources, particularly notable for their high potassium and low sodium contents—an advantageous feature for blood pressure regulation [6].

From a functional standpoint, potassium contributes to electrolyte homeostasis and endothelial-dependent vasodilation, magnesium serves as a cofactor in phosphorylation reactions (e.g., glycolysis and ATP synthesis), phosphorus plays a structural role in phospholipids and bone while participating in energy metabolism, and iron and zinc support oxygen transport and enzymatic immune function, respectively. Low sodium content further reinforces the relevance of *mashua* in cardioprotective dietary patterns. The high global disease burden associated with iron deficiency in vulnerable populations underscores the importance of maintaining adequate iron and trace element intake, highlighting the value of complementary plant matrices within diversified diets [2,4].

### 5.3. Bioactive Compounds

*Mashua* is rich in polyphenols, flavonoids, and anthocyanins—compounds responsible for its high antioxidant capacity (Table 4). Purple-colored varieties exhibit the highest concentrations, making them particularly valuable for health promotion and disease prevention [34]. In addition, *mashua* contains thiocyanates, glucosinolates, isothiocyanates, and carotenoids, such as lutein and β-carotene, which contribute to its antioxidant properties and health benefits [3].

Table 4 presents the total anthocyanin, flavonoid, and phenolic contents of *mashua* (dry weight basis) across different varieties, ecotypes, and accessions. These data emphasize that *mashua* stands out not only for its nutritional composition but also for its richness in bioactive compounds responsible for its antioxidant activity and potential role in disease prevention [6]. Pigmented accessions, particularly purple varieties, contain higher concentrations of these phytochemicals, suggesting promising applications as functional or nutraceutical ingredients in the food industry [4,25]. These findings highlight the importance of conserving the genetic diversity of this ancestral crop and promoting its consumption for its nutritional value and therapeutic potential.

#### 5.3.1. Polyphenols

*Mashua* exhibits marked variability in its phenolic content, strongly influenced by genotype, pigmentation, and agroecological conditions. In three contrasting genotypes from Puno (purple, yellow, and yellow–purple flesh), total phenolics ranged from 1.16 to 11.43 mg GAE/g DW, with consistently higher values in purple morphotypes and significant differences attributed to both genotype and environment [4]. Similarly, in a comparative study of three genotypes with different pigmentation, purple tubers showed higher phenolic contents (8.63–11.04 mg GAE/g DW) than yellow ones (~4.54 mg/g DW), correlating positively with greater antioxidant capacity determined by ORAC, DPPH, and FRAP assays [38].

The superiority of purple morphotypes is largely explained by their anthocyanin fraction. Total anthocyanin contents have been reported to be between 0.5 and 2.05 mg cyanidin-3-glucoside equivalents per gram of fresh sample (C3G/g FW), predominantly in purple-fleshed ecotypes, which also display complex profiles of anthocyanidins and proanthocyanidins [6]. Anatomically, the peel contains higher polyphenol concentrations than the flesh: 27.22 mg GAE/g DW in the flesh and 28.37 mg GAE/g DW in the peel, confirming that the outer fraction possesses the greatest “composite antioxidant potency” among Andean tubers [39].

Genetic variability is also a key determinant. The total phenolic content of 27 Peruvian morphotypes varied widely from 2990.8 ± 273.5 to 24,217.4 ± 1144 mg/kg DW, highlighting the importance of germplasm as a reservoir of bioactive compounds. Moreover, postharvest application of methyl jasmonate increased polyphenol levels by up to 150% [40]. Regionally, Ecuadorian varieties such as *Izaño* displayed high antioxidant capacity attributed to their polyphenolic fraction, together with carotenoids and vitamin C [10], while metabolomic analyses using nuclear magnetic resonance (NMR) confirmed significant differences in metabolite profiles among ecotypes [13].

Recent studies in Bolivia have shown that purple *isaño* (*mashua*) cultivars contain elevated levels of flavonoids and anthocyanidins, which are associated with higher antioxidant activity [3]. In Ecuador, purple *mashua* was reported to contain up to 450 ± 7 mg GAE/100 g DW, surpassing other Andean tubers [22].

Chromatographic analyses have detailed the individual phenolic composition: flavan-3-ols such as (–)-epicatechin (9.2 µg/g DM), flavonols such as quercetin-3-O-rutinoside (40.6 µg/g DM), kaempferol derivatives up to 46.2 µg/g DM, and phenolic acids, including protocatechuic and caffeic acids, have been identified [31,41]. Ethanolic extracts of *mashua* demonstrated antioxidant activity superior to BHT in inhibiting lipid oxidation in vegetable oils [15].

Processing also modulates the phenolic profile. Microwave blanching (MWB) prior to drying showed significant advantages, increasing phenolics by 11.6%, anthocyanins by 24.8%, and carotenoids by 87.4% before dehydration; after drying, samples retained 532.4 mg GAE/100 g DW with high antioxidant capacity (94.4% DPPH), outperforming hot-water or ultrasound blanching [42].

Accumulated evidence confirms that *mashua* is one of the Andean tubers rich in phenolic compounds, with purple morphotypes and peel fractions representing matrices of high bioactive value. However, the following methodological limitations hinder inter-study comparisons: lack of standardization in reporting basis (fresh vs. dry weight), use of different equivalents (GAE, CE, C3G), and scarce information on in vivo bioavailability and metabolic kinetics. Although emerging technologies such as microwave blanching show promise for preserving or enhancing phenolics, pilot-scale or clinical trials have not yet validated their functional effects. Future research should prioritize standardized protocols and bioaccessibility studies, integrating omics techniques and clinical assays to consolidate *mashua* as a functional ingredient and natural antioxidant in the food industry.

#### 5.3.2. Flavonoids

Flavonoids constitute a relevant subgroup within the *mashua* phenolic profile. The compounds identified include catechin, epicatechin, gallocatechin, quercetin, and their glycosylated derivatives [31,41].

When quantified directly in flour (DW basis), total flavonoids are relatively low (≈0.027–0.164 mg CE/g DW) and depend on genotype and environment, with lower contents in yellow–purple morphotypes (0.027 ± 0.0004 to 0.086 ± 0.001 mg/g DW) [4]. However, anatomical fractionation revealed that the peel concentrates more flavonoids than the flesh and exhibits greater antioxidant capacity, reinforcing the functional value of colored by-products as natural ingredients [6,39].

Flavonoid accumulation varies with tuber pigmentation: purple accessions contain up to eight times more total flavonoids (77–79 mg CE/100 g FW) than yellow ones (<10 mg CE/100 g FW), establishing a direct relationship between color intensity, flavonoid composition, and antioxidant capacity [38]. The total flavonoid content ranges from 10.9 to 45.3 mg quercetin equivalents/100 g DW, with the highest concentrations found in the purple ecotypes [42].

Specific anthocyanins such as delphinidin and cyanidin in acylated forms confer the intense pigmentation and high antioxidant activity characteristic of purple tubers [3,36]. These flavonoids (ORAC and FRAP) correlate positively with radical-scavenging capacity, suggesting a direct role in *mashua*’s nutraceutical potential [38].

*Mashua* contains appreciable amounts of flavonoids, mainly flavonols and flavan-3-ols. Notable compounds include quercetin-3-O-rutinoside (40.6 µg/g DM), isorhamnetin-3-rutinoside (6.9 µg/g DM), myricetin dihexoside and acetylated derivatives, epicatechin (9.2 µg/g DM), and epigallocatechin [31]. These compounds contribute not only to antioxidant activity but also exhibit anti-inflammatory, anticarcinogenic, and cardioprotective properties.

#### 5.3.3. Glucosinolates (GLS) and Isothiocyanates

*Mashua* exhibits a profile dominated by aromatic glucosinolates (Table 5), mainly *p*-hydroxybenzyl (OHB, glucosinalbin), benzyl (B, glucotropaeolin), and *p*-methoxybenzyl (MOB, glucoaubrietin) [43]. The total contents vary widely among accessions, ranging from 0.27 to 50.74 µmol/g DW in materials from Cusco [43], with “sweet *mashua*” types showing <5 µmol/g, predominant among cultivated populations, and some feral lines [43]. In Colombia, values between <0.3 and 25.8 µmol/g DW have been quantified, with *p*-methoxybenzyl glucosinolate as the predominant compound [44].

The hydrolysis of glucotropaeolin and other derivatives by myrosinase during processing or mastication generates isothiocyanates (ITCs), which are responsible for the characteristic pungent flavor and the bioactivity of *mashua*. Reported ranges extend from approximately 0.27 to >50 µmol/g DW, with even higher concentrations in specific varieties, differences mainly attributed to genotype, postharvest handling, and analytical methods [6]. The main hydrolysis product in *T. tuberosum* subsp. tuberosum is *p*-methoxybenzyl isothiocyanate (p-MBITC), while benzyl-, 2-propyl-, and 2-butyl-ITCs are also released in wild subspecies. Additionally, N,N-di(4-methoxybenzyl)thiourea has been detected as a derivative in tuber [45]. These metabolites play ecological roles in plant defense and are nutritionally associated with antimicrobial and chemopreventive activities. However, their bioavailability and risk–benefit balance strongly depend on processing conditions (e.g., cooking, light exposure, and fermentation), which typically reduce the levels of non-toxic sulfur-containing compounds such as thiocyanates [6].

During postharvest storage and processing, refrigeration (12 °C; 80% RH) followed by drying leads to significant GLS losses (−38% to −87% relative to the control), accompanied by the detection of degradation products such as p-MBITC, 4-methoxybenzeneacetonitrile, 4-methoxybenzyl alcohol-ME, BITC, and benzeneacetaldehyde [46]. The total GLS (tGLS) in the flours produced after postharvest decreased from 26.6 ± 0.7 to 3.6 ± 0.1 µmol/g DW by day 12, with poor correlation between these losses and myrosinase activity [46]. This behavior suggests that non-enzymatic cofactors (e.g., ESP/NSP-type proteins) modulate the GLS→ITC transformation during postharvest handling [46].

From an applied perspective, the presence and concentration of aromatic GLS in *mashua* support its potential as a biofumigant and biological control agent in agriculture, although the authors emphasize the need for in vivo assays to validate the efficacy and field conditions [44].

Overall, *mashua* represents an exceptional source of aromatic GLS, ranging from 0.27 to 50.74 µmol/g DW (and up to 25.8 µmol/g DW in Colombian accessions), and releases ITCs of high interest such as p-MBITC and BITC. Nevertheless, genotype heterogeneity, instability during drying, and the weak correlation between myrosinase activity and real GLS losses highlight the urgent need to: (1) standardize postharvest protocols (particularly drying) to minimize degradation; (2) model the GLS→ITC conversion kinetics incorporating cofactors (ESP/NSP) in real matrices; and (3) translate current findings to in vivo and field studies including dose–response metrics and metabolic fate of ITCs. These steps are essential for transforming *mashua* into reproducible and scalable functional and agronomic applications.

**Table 5:** Total glucosinolate content (dry weight basis) of *T. tuberosum* according to variety, ecotype, and accession.

Variety/Ecotype/Accession	Total Glucosinolates (µmol/g Dry Weight)	Reference
Yellow–Yellow	0.81–9.53	[44]
White–Purple	0.97–10.30	
White–Red	0.85–25.8	
Purple–Purple	1.18–7.75	
ARB 5241	8.20	[47]
AGM 5109	7.50	
DP 0224	9.00	
DP 0207	5.50	
M6COL2C	6.80	
DP 0215	7.00	
Black *mashua*	36.50	[17]
Yellow *mashua*	90.00	
Red *mashua*	50.74	
Kellu (Peru)	29.7	[11]
Chejchi (Peru)	46.5	
Chiar (Bolivia)	4.4	
Kellu (Bolivia)	18.3	
Keni Kellu (Bolivia)	14.5	
Jachir (Bolivia)	63.5	
Asuthi (Bolivia)	35.7	
ARB 5241 (purple)	54.2 ± 4.9	[48]
DP 0224 (purple)	51.2 ± 4.3	
Chiara (dark)	48.0 ± 0.4	
*Isaño* 02	32.4 ± 1.4	
Zapallo	29.4 ± 1.0	
K’ello 01	38.7 ± 5.9	
K’ello 19	12.9 ± 0.8	
*Isaño* 20	19.9 ± 2.5	
Wild *mashua*	0.27–50.74	[16]
Cultivated *mashua*	0.3–25.8	
Purple *mashua* varieties	4.9–54.2	[46]
Wild *mashua* varieties	0.27–50.74	
Cultivated	0.3–25.8	

#### 5.3.4. Antioxidant Capacity

*Mashua* is widely recognized as one of the Andean tubers with the highest antioxidant capacity (Table 6), mainly attributed to its high concentration of phenolic compounds, flavonoids, and anthocyanins. The *mashua* shows clear and consistent differences among morphotypes, with purple and black varieties exhibiting markedly higher values than yellow or cream types. Comparative studies have shown that purple tubers exhibit antioxidant capacities 8–10 times greater than yellow tubers, with anthocyanin contents ranging from 45.5 to 131.9 mg cyanidin-3-glucoside equivalents/100 g fresh weight, levels comparable to those reported for blueberries and blackcurrants [49].

Free radical–scavenging assays confirmed this strong antioxidant activity. The total phenolic content of *mashua* ranges from 14.4 to 18.7 mg GAE/g DW, with ORAC values between 221 and 359 µmol Trolox equivalents (TE)/g DW [50]. On a fresh weight basis, reported values range from 174.9 to 275.5 mg GAE/100 g and 16.2 to 45.7 µmol TE/g [49]. In purple genotypes, ORAC values of 273–379 µmol TE/g DW have been reported, far exceeding those of well-known antioxidant fruits such as blackberry, raspberry, and strawberry (35–162 µmol TE/g DW) [51].

Moreover, purified phenolic extracts have demonstrated protective effects on oxidation-sensitive biological structures. In vitro assays showed that TBARS and conjugated diene formation inhibited LDL oxidation by 29.1–34.8% and 51.8–58.1%, respectively, as well as erythrocyte hemolysis by 20.8–25.1% [51]. The positive correlation between polyphenol/flavonoid levels and low-density lipoprotein (LDL) protection underscores the physiological relevance of these metabolites.

In food matrices, *mashua* extracts, especially those enriched in phenolics, demonstrate strong antioxidant efficacy. Ethyl acetate–soluble fractions, containing 22.2 mg GAE/mL and 200.2 µmol TE/mL of antioxidant activity, significantly delayed lipid oxidation in *sacha inchi* oil and minced pork, showing a stronger effect than the synthetic antioxidant BHT [18]. Similarly, the addition of *mashua* extracts to soybean oil during frying reduced the formation of dimer and trimer, demonstrating higher oxidative stability than oil supplemented with TBHQ [52].

Recent research has highlighted the variability in antioxidant activity depending on genotype and processing conditions. In black *mashua*, antioxidant values reached up to 446.7 µmol TE/g, although anthocyanin bioaccessibility remained low (22.4%), and processes such as blanching reduced pigment levels by up to 73% [35]. Comparative evaluations of Andean roots and tubers identified *mashua* peel as having the highest APC index, surpassing *oca* and *ulluco* [53].

Technological strategies, such as microencapsulation, have proven to be effective in preserving the antioxidant capacity of *mashua.* Spray drying with modified *oca* and *ulluco* starches resulted in powders with encapsulation efficiencies above 70% and antioxidant capacities up to 42.9 µmol TE/g, confirming their potential as stable functional ingredients [54].

**Table 6:** Antioxidant capacity of *T. tuberosum* evaluated by DPPH, ABTS, FRAP, and ORAC according to variety and analyzed material.

Variety/Accession	Material and Preparation	DPPH	ABTS	FRAP	ORAC	Ref.
Tt-23 (P/P)	Whole tuber (FW)	68.25 ± 1.80 µM TEAC/100 g	n.d.	2299.03 ± 25.46 mM Fe^2+^/100 g	n.d.	[38]
Tt-03 (purple)	Whole tuber (FW)	60.84 ± 1.53 µM TEAC/100 g	n.d.	1242.52 ± 16.67 mM Fe^2+^/100 g	n.d.	
Tt-25	Whole tuber (FW)	60.28 ± 1.10 µM TEAC/100 g	n.d.	1055.53 ± 4.66 mM Fe^2+^/100 g	n.d.	
Tt-02	Whole tuber (FW)	43.00 ± 1.44 µM TEAC/100 g	n.d.	985.63 ± 4.62 mM Fe^2+^/100 g	n.d.	
Tt-19	Whole tuber (FW)	35.26 ± 1.08 µM TEAC/100 g	n.d.	460.78 ± 4.20 mM Fe^2+^/100 g	n.d.	
Tt-11	Whole tuber (FW)	28.35 ± 0.65 µM TEAC/100 g	n.d.	390.68 ± 14.30 mM Fe^2+^/100 g	n.d.	
ARB-5241 (P/Y)	Whole tuber (DW)	n.d.	80–378 µmol TE/g	n.d.	271–446 µmol TE/g	[34,36]
DP-0224 (P/P)	Whole tuber (DW)	n.d.	80–378 µmol TE/g	n.d.	221–359 µmol TE/g	[34,36,41]
AGM-5109 (P/Y)	Whole tuber (DW)	n.d.	80–378 µmol TE/g	n.d.	271–446 µmol TE/g	[34,36]
ARB-5576 (Y/Y)	Whole tuber (DW)	n.d.	80–378 µmol TE/g	n.d.	59–389 µmol TE/g	[36,41]
AVM-5562 (Y/Y)	Whole tuber (DW)	n.d.	80–378 µmol TE/g	n.d.	59–389 µmol TE/g	[36]
M6COL2C (Y/Y)	Whole tuber (DW)	n.d.	80–378 µmol TE/g	n.d.	59–389 µmol TE/g	
DP-0203 (Y/Y)	Whole tuber (DW)	n.d.	80–378 µmol TE/g	n.d.	59–389 µmol TE/g	
DP-0223 (Y/Y)	Whole tuber (DW)	n.d.	80–378 µmol TE/g	n.d.	59–389 µmol TE/g	
DP-0207 (Y/Y)	Whole tuber (DW)	n.d.	80–378 µmol TE/g	n.d.	59–389 µmol TE/g	
DP-0215 (Y/Y)	Whole tuber (DW)	n.d.	80–378 µmol TE/g	n.d.	59–389 µmol TE/g	
ARV-5366 (Y–P/P)	Whole tuber (FW)	n.d.	3.82–39.15 µmol TE/g	n.d.	n.d.	[34]
DP-0224 (P/P)	Whole tuber (FW)	n.d.	3.82–39.15 µmol TE/g	n.d.	n.d.	
*Mashua*	Separated pulp (FW)	172.01 ± 3.41 µmol TE	274.81 ± 6.49 µmol TE	226.47 ± 0.37 µmol TE	n.d.	[53]
*Mashua*	Separated peel (FW)	309.62 ± 0.43 µmol TE	335.49 ± 9.48 µmol TE	570.95 ± 4.47 µmol TE	n.d.	
Yellow *mashua*	Whole tuber (DW)	n.d.	n.d.	n.d.	76.43 µmol Trolox/g	[55]
Yellow *mashua*	Whole tuber–SCF extract (DW)	n.d.	n.d.	n.d.	5732.10 µmol Trolox/g	
White *mashua*	Whole tuber (DW)	n.d.	n.d.	n.d.	101.66 µmol Trolox/g	
White *mashua*	Whole tuber–SCF extract (DW)	n.d.	n.d.	n.d.	6596.10 µmol Trolox/g	
Pink *mashua*	Hydroalcoholic extract (50 µg/mL)	19.82 ± 2.01%	21.10 ± 1.54%	66.38 ± 3.85 µM AA eq	n.d.	[12]
Pink *mashua*	Hydroalcoholic extract (250 µg/mL)	63.10 ± 2.48%	70.17 ± 1.40%	115.27 ± 15.12 µM AA eq	n.d.	
Yellow *mashua*	Hydroalcoholic extract (50 µg/mL)	31.99 ± 3.48%	33.89 ± 1.76%	74.16 ± 6.66 µM AA eq	n.d.	
Yellow *mashua*	Hydroalcoholic extract (250 µg/mL)	75.45 ± 1.54%	76.48 ± 1.35%	270.83 ± 12.58 µM AA eq	n.d.	
Black *mashua*	Hydroalcoholic extract (50 µg/mL)	47.37 ± 3.96%	41.87 ± 1.79%	74.72 ± 9.18 µM AA eq	n.d.	
Black *mashua*	Hydroalcoholic extract (250 µg/mL)	83.81 ± 0.86%	87.86 ± 1.76%	474.71 ± 15.03 µM AA eq	n.d.	
Wild purple *isaño*	Whole tuber (DW)	405.45 ± 0.94 µmol TE/g	n.d.	n.d.	n.d.	[23]
Wild yellow *isaño*	Whole tuber (DW)	115.36 ± 0.77 µmol TE/g	n.d.	n.d.	n.d.	
Wild pink *isaño*	Whole tuber (DW)	32.18 ± 0.14 µmol TE/g	n.d.	n.d.	n.d.	
*Mashua*	Whole tuber (FW)	15.80 ± 0.2 µmol TE/g	17.0 ± 0.2 µmol TE/g	n.d.	n.d.	[56]
*Mashua*	Whole tuber –ethanolic extract	n.d.	2.18 ± 0.25 µg GAE/mg	n.d.	n.d.	[57]
ARB 5241	Phenolic fraction I (FW)	n.d.	n.d.	n.d.	1.2–6.5 µmol TE/g	[41]
ARB 5241	Phenolic fraction II (FW)	n.d.	n.d.	n.d.	0.2–2.7 µmol TE/g	
ARB 5241	Phenolic fraction III (FW)	n.d.	n.d.	n.d.	1.9–17.1 µmol TE/g	
ARB 5241	Phenolic fraction IV (FW)	n.d.	n.d.	n.d.	3.1–21.0 µmol TE/g	
ARB 5576	Phenolic fraction I (FW)	n.d.	n.d.	n.d.	6.5 µmol TE/g	
ARB 5576	Phenolic fraction II (FW)	n.d.	n.d.	n.d.	2.7 µmol TE/g	
ARB 5576	Phenolic fraction III (FW)	n.d.	n.d.	n.d.	17.1 µmol TE/g	
ARB 5576	Phenolic fraction IV (FW)	n.d.	n.d.	n.d.	21.0 µmol TE/g	
DP 0224	Phenolic fraction I (FW)	n.d.	n.d.	n.d.	4.2 µmol TE/g	
DP 0224	Phenolic fraction II (FW)	n.d.	n.d.	n.d.	2.6 µmol TE/g	
DP 0224	Phenolic fraction III (FW)	n.d.	n.d.	n.d.	17.6 µmol TE/g	
DP 0224	Phenolic fraction IV (FW)	n.d.	n.d.	n.d.	5.8 µmol TE/g	
Various genotypes	Whole tuber (DW)	n.d.	16.2–45.7 µmol TE/g	n.d.	–	[49]
*Mashua* (ethanolic extract)	Whole tuber (DW)	1.2–812.8 µmol TE/g	3.7–1045.3 µmol TE/g	n.d.	6.5–2326.2 µmol TE/g	[58]
*Mashua* (ethyl acetate fraction [EaF])	Whole tuber–ethyl acetate fraction	–	200.2 µmol TE/mL	–	–	[18]
INIAP-ECU *Izaño*	Whole tuber (DW)	57.2 mM Trolox/100 g	–	–	36.7 ± 1.6 µmol TE/g	[10,35]
Purple *mashua* (*PME* powder)	Whole tuber—freeze-dried powder	18.07–47.83 µmol Trolox/g	–	–	–	[54]
Black *mashua* (optimized extract)	Whole tuber–optimized hydroalcoholic extract	0.275 ± 0.003 mM Trolox eq/mL	–	–	–	[26]
Fresh *mashua* (Yunguyo and Puno)	Whole tuber (FW)	2221.02 ± 2.0 µM Trolox eq/100 g	–	–	–	[26]
*Santo Jonk’ori* (Bolivia)	Whole tuber (FW)	–	2.9 ± 0.6 µmol TE/g	4.5 ± 0.5 µmol TE/g	–	[3]
*Chakari* (Bolivia)	Whole tuber (FW)	–	4.2 ± 0.7 µmol TE/g	7.0 ± 0.9 µmol TE/g	–	
*Ch’iyara* (Bolivia)	Whole tuber (FW)	–	11.6 ± 0.9 µmol TE/g	30.0 ± 1.5 µmol TE/g	–	

Abbreviations: FW: fresh weight; DW: dry weight; P/P: purple skin/purple flesh; P/Y: purple skin/yellow flesh; Y/Y: yellow skin/yellow flesh; SCF: supercritical fluid extraction; TE: Trolox equivalents; TEAC: Trolox equivalent antioxidant capacity; GAE: gallic acid equivalents; AA eq: ascorbic acid equivalents. Phenolic fractions I–IV refer to HPLC-purified subfractions from whole tubers; anthocyanins typically show the highest antioxidant activity in fraction III. Hydroalcoholic extracts (50–250 µg/mL) derived from dehydrated, powdered tubers correspond to final extract concentrations used in antioxidant assays. Approximate conversion: 1 g DW ≈ 3–5 g FW (considering 70–80% moisture in fresh *mashua*). The fresh and dry weight values are not directly comparable without conversion. The extraction methods (conventional, ultrasound, and SCF) significantly affect the yield variability. n.d.: not determined.

Overall, the consistently superior antioxidant performance of purple and black *mashua* varieties highlights a clear morphotype-dependent trend: darker tubers exhibit markedly higher phenolic content, anthocyanin concentration, and ORAC values than yellow or cream tubers. However, the full exploitation of high antioxidant morphotypes remains limited by genotype variability, significant pigment degradation during thermal processing, and low anthocyanin bioaccessibility. Therefore, future studies should prioritize standardizing analytical methodologies, evaluating In Vivo bioavailability, and applying emerging technologies to enhance the stability and functionality of its metabolites.

Only through these efforts can *mashua* be consolidated as a strategic source of natural antioxidants for the nutraceutical and food industries.

#### 5.3.5. Other Components

In addition, *mashua* contains metabolites of functional interest to polyphenols, flavonoids, and glucosinolates. In Bolivian and Peruvian cultivars, carotenoids such as lutein, neoxanthin, and β-carotene have been identified, with reported concentrations ranging from 1 to 25 µg β-carotene/g fresh weight [34]. More recent studies found that the total carotenoid content among 27 Peruvian morphotypes varied from 12.8 to 85.8 mg/kg DW, and postharvest treatment with methyl jasmonate increased carotenoid concentrations by up to 535% compared with the control, indicating a co-regulated isoprenoid/phenylpropanoid metabolism [40].

Methanolic extracts of *mashua* contain tocopherols and phytosterols (γ-tocopherol, campesterol, and β-sitosterol), as well as alkamides with bioactive potential, contributing to protection against oxidative stress and exhibiting antimicrobial effects [42,55].

Regarding tannins and flavan-3-ols, a subfraction relevant for their protein–tannin interactions during digestion, reported levels range from 0.2 to 5.3 mg catechin equivalents/g DW, with a high proportion of condensed tannins in *mashua*, which display biphasic nutritional effects (moderate doses: potential protein protection benefits; high doses: reduced digestibility) [6]. Sulfur-containing derivatives, such as 4-methoxybenzyl isothiocyanate, complement the functional profile described for glucosinolates and reinforce the species’ chemoprotective potential [55]. In addition, compounds with insecticidal activity derived from glucosinolates have been described, highlighting the multifunctional nature of *mashua* for human nutrition and sustainable agricultural applications [59].

## 6. Potential Food Applications of *Mashua*

The diverse biochemical profile of *mashua*, characterized by its richness in starch, essential nutrients, and phytochemicals such as glucosinolates, anthocyanins, and carotenoids, provides a foundation for its use in functional and technologically enhanced food systems. Current research explores its incorporation into bakery products, extruded snacks, dairy matrices, and fermented beverages as part of strategies to develop nutritious and sustainable foods [10,20,60].

### 6.1. Bakery and Snacks

The use of *mashua* flour in baking and gluten-free products has shown promising results. In muffins formulated with *mashua*, oca, and sweet potato blends, the protein and fiber contents reached 9.12% and 9.6%, respectively, which were higher than those of wheat flour (10.2% protein, 2.7% fiber), with good sensory acceptability, particularly in sucralose-sweetened formulations. Sun exposure enhances the conversion of starch to simple sugars, allowing the reduction or replacement of added sucrose [47].

*Mashua* is also suitable for developing second- and third-generation extruded snacks. Microwave expansion preserved bioactives and sensory attributes in 80:20 corn/ *mashua* pellets [29]. The addition of *mashua* to second-generation snacks increased carotenoids and phenolics, positioning these products as functional foods [28]. Additionally, *mashua* chips processed by vacuum frying showed 50.42% less fat than those processed by atmospheric frying while maintaining crispness [61].

### 6.2. Derivatives of Yogurt and Dairy Products

Incorporating *mashua* into dairy products targets both fermentation and natural coloring. Oat extracts with *mashua* pulp fermented with lactic cultures reached >10^6^ CFU/g, meeting probiotic thresholds, with an acceptable sensory profile [62]. Optimized extracts from black *mashua* provided anthocyanins at 347.34–419.92 mg/L and polyphenols at 63.68–105.09 mg/100 g, with an antioxidant capacity of 0.275 ± 0.003 mM Trolox eq./mL. Yogurts containing 6% extract exhibited the highest sensory acceptability [26].

### 6.3. Alcoholic and Fermented Beverages

*Mashua* has been evaluated to produce wines and artisanal beverages. In “*isaño* wine” (a *mashua* variety), ultrasound treatment (100 W, 84 h) increased ethanol yield to 10.36% *v/v* in purple genotypes [63]. In Colombia, a *cubio* (*mashua*) fermented beverage reached 15.24 Alcohol by Volume (ABV) with adequate sensory acceptance, demonstrating technological and commercial feasibility [64]. These results indicate that competitive products can be obtained by enzymatic or physical pretreatments, although glucosinolates may interfere with fermentation.

### 6.4. Other Foods and Contemporary Gastronomy

In gourmet cuisine, *mashua* is valued for its versatility and nutraceutical properties and is used in high-end restaurants in Peru, Bolivia, and Ecuador [65]. Archaeobotanical studies have confirmed its consumption in Quito for more than 2500 years [66], reinforcing its cultural importance and potential for revalorization. Its profile of antioxidants, carotenoids, and glucosinolates supports its application as a functional ingredient in modern food matrices [8,10].

Despite extensive experimental evidence supporting the feasibility of using *mashua* in baked goods, extruded products, dairy matrices, and fermented beverages, significant limitations remain. Most studies report laboratory-scale results with limited information on storage stability, consumer acceptance in broader markets, or cost–benefit analyses. Moreover, the pungent flavor and genotypic variation in glucosinolate content continue to hinder industrial adoption. Therefore, technological optimization studies (debittering processes, bioactive microencapsulation, and green fermentation technologies) should be integrated with market analyses and pilot-scale upscaling. Only then can *mashua* be consolidated not only as an ancestral resource of cultural value but also as a competitive ingredient in the global functional foods industry.

## 7. Traditional Uses of the *mashua*

*Mashua* has been integral to Andean food and healing traditions for centuries. Its continued cultivation in Peru, Bolivia, Ecuador, and Colombia reflects the preservation of ancestral knowledge that recognizes its nutritional value and therapeutic potential [8,63].

### 7.1. Ancestral Food Use

Historically, *mashua* was consumed boiled, roasted, or stewed, often in combination with other native tubers and vegetables, such as *oca* and *ulluco* [8]. Archaeobotanical studies in Quito revealed *mashua* starch granules in ceramic utensils dating back over 2500 years, confirming its integration into pre-Hispanic diets [66]. Consumption was linked to communal and festive preparations, serving as an energy source at altitudes above 3000 m a.s.l., where other crops had limited productivity [20].

### 7.2. Medicinal and Ethnobotanical Uses

In traditional medicine, *mashua* has been used as a diuretic, anti-inflammatory, hepatoprotective, and nephroprotective agent [63]. Isothiocyanates derived from its glucosinolates have been associated with antimicrobial and anticancer properties, supporting its empirical use for prostate, skin, and urinary disorders [8,10]. In Ecuadorian and Peruvian communities, *mashua* has also been used as a topical skin poultice and to treat respiratory ailments [62].

### 7.3. Cultural and Culinary Values

Beyond its therapeutic role, the *mashua* carries deep cultural significance. It has been traditionally attributed with anaphrodisiac effects; hence, it is recommended for monks and soldiers to suppress sexual desire [14]. In recent times, its use in soups, stews, and artisanal fermented foods has persisted in rural areas, though less frequently than other tubers such as potato and maize [65]. In Peru and Bolivia, gourmet restaurants have reintroduced *mashua* as an emblematic ingredient of modern haute cuisine in the Andes [65].

The traditional uses of *mashua* reflect a profound ancestral knowledge that integrates dietary, medicinal, and cultural aspects. However, many of these practices remain poorly documented and orally transmitted, limiting their scientific validation and modern application. Although phytochemical studies partially support the ethnomedical properties of glycosinolates, polyphenols, and anthocyanins, a gap remains in the clinical systematization and preservation of traditional culinary knowledge. A critical review underscores the need to integrate ethnographic studies with biomedical and technological research to preserve and validate the cultural heritage of the *mashua* people while maintaining their ancestral identity and fostering their acceptance in modern markets.

Although the available literature provides valuable insights into *mashua* composition and antioxidant behavior, several methodological limitations affect the comparability and interpretation of results. Analytical approaches used to quantify phenolics, glucosinolates, anthocyanins, and antioxidant activity, including differences in extraction solvents, particle size, sample pretreatment, and assay conditions, vary widely among studies. The heavy reliance on chemical antioxidant assays (DPPH, ABTS, FRAP, and ORAC), each with different reaction mechanisms and sensitivities, complicates direct comparison across research groups and hampers the establishment of standardized reference values. Furthermore, advanced metabolomic and proteomic analyses remain limited, limiting our understanding of genotype–metabolite interactions and the biochemical basis of morphotype variability. Small sample sizes, heterogeneous genetic materials, and non-uniform postharvest handling further weaken reproducibility. These factors highlight the need for more harmonized analytical methodologies and comprehensive multiomic approaches in future *mashua* research.

## 8. Conclusions and Future Prospects

*Mashua* stands out as a native Andean tuber with remarkable nutritional and functional potential, characterized by its high starch content, vitamin C, glucosinolates, and phenolic pigments. Its starch content exhibits low gelatinization temperature and limited retrogradation, broadening its technological applicability in food and biopolymeric matrices. Processing operations, such as cooking and fermentation, significantly reduce the amount of sulfur compounds responsible for the pungent flavor, thereby enhancing both safety and sensory acceptability.

Current evidence supports the feasibility of *mashua* in various food products, baked goods, extruded snacks, low-fat chips, yogurts enriched with anthocyanin extracts, and fermented beverages, all showing promising physicochemical and sensory performance. Nevertheless, most research remains at the laboratory scale, with limited industrial validation, scarce data on shelf-life stability, and insufficient cost–benefit analyses. Genotypic variability in glucosinolate and anthocyanin content continues to be a major challenge for product standardization.

**Figure 3:**
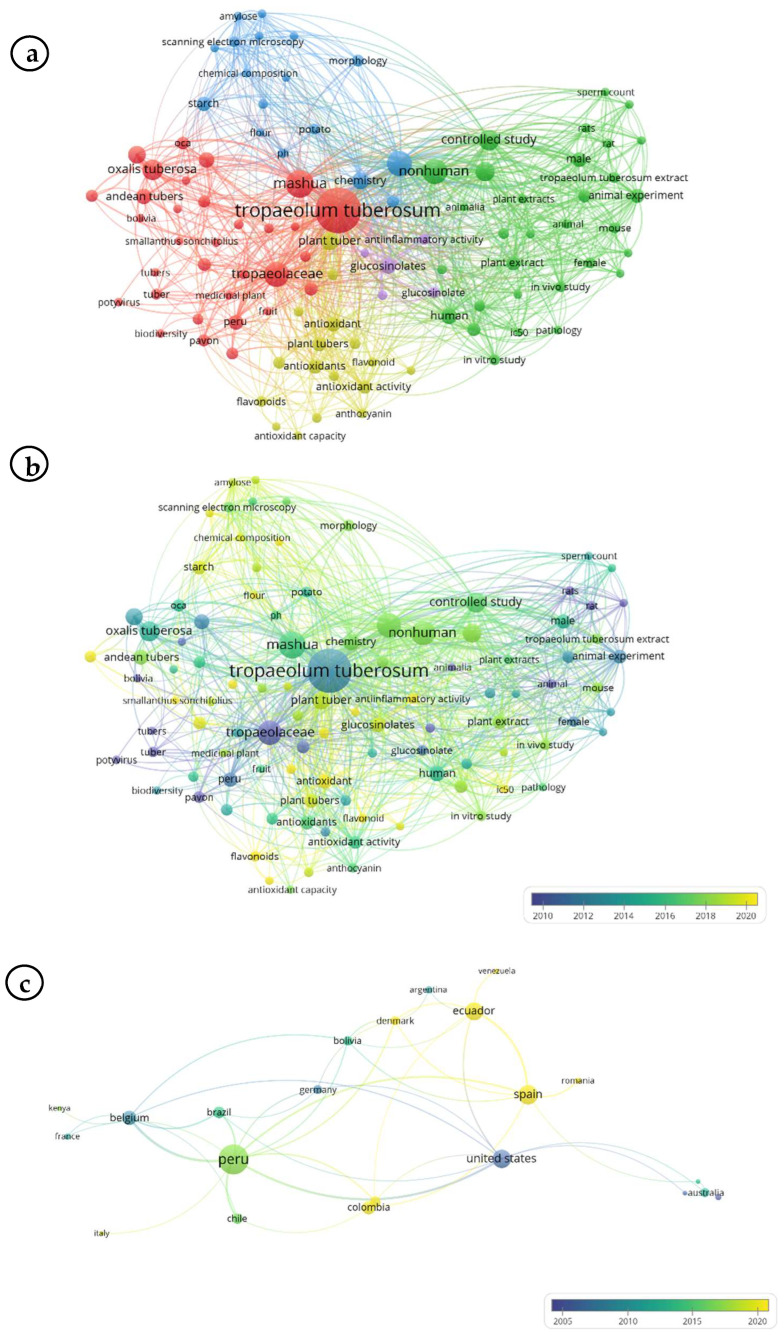
Bibliometric visualization maps on *T. tuberosum* generated using VOS viewer (version 1.6.20) from a Scopus search conducted in October 2025 with the query TITLE-ABS-KEY (“*mashua*” OR “*T. tuberosum*”): (**a**) Keyword co-occurrence clustering; (**b**) temporal evolution of keyword co-occurrence (2010–2020); (**c**) international co-authorship network by country (2005–2020).

The bibliometric analysis (Figure 3) revealed that *T. tuberosum* research has focused primarily on chemical composition, antioxidant capacity, and bioactive compounds, particularly glucosinolates, flavonoids, and anthocyanins. Thematic clustering (Figure 3a,b) highlights four dominant research fronts, namely, chemical composition, antioxidant activity, glucosinolates, and Andean tubers, while the international co-authorship network (Figure 3c) provides evidence of active collaboration among Peru, Ecuador, Spain, Belgium, and the United States. The growing number of publications since 2015 underscores an expanding global interest in this underutilized crop and its potential applications in functional food systems.

Recent advances suggest that integrating multi-omics approaches (proteomic and metabolomic) is crucial for linking genetic diversity with secondary metabolite biosynthesis [40,67]. Emerging non-thermal technologies, such as ultrasound, high hydrostatic pressure, pulsed electric fields, and methyl-jasmonate treatments, represent promising tools for enhancing bioactive accumulation and extending postharvest stability [40,68]. Future research should focus on standardizing postharvest handling, optimizing green extraction techniques, and validating the bioefficacy of *mashua*-derived compounds through in vivo and clinical studies.

Altogether, *mashua* embodies a bridge between ancestral biodiversity and modern innovation. By integrating advanced technologies such as microencapsulation, active biopolymer films, and 3D food printing, this Andean crop can evolve from a traditional food source into a sustainable, high-value functional ingredient with strong cultural identity and global potential in the nutraceutical and food industries.

## Figures and Tables

**Table 1:** Morphological and Taxonomic Characteristics of *T. tuberosum*.

Aspect	Details	References
Taxonomy	*Tropaeolum tuberosum* belongs to the family Tropaeolaceae. Classification: Kingdom Plantae, Magnoliophyta, Magnoliopsida, Brassicales, Tropaeolum, and *T. tuberosum*.	[2,14,15]
Common Names	*Mashua* in Peru, *isaño* in Bolivia, *cubio* in Colombia, and *añu* in Ecuador.	[3,14,16]
Morphological Characteristics	Perennial herbaceous plant producing multicolored conical tubers (yellow, red, purple, and black). The tubers have smooth and irregular surfaces. The leaves are rounded with slightly wavy margins.	[3,17,18]
Ecological Distribution	Native to the Andes, cultivated between 1500 and 4300 m above sea level. Its main distribution occurs in Peru, Bolivia, Ecuador, and Colombia.	[3,14]
Preferred Climate	Cool temperate; tolerant to light frosts and short heat peaks up to 30 °C. Optimal in areas with annual precipitation between 700 and 1400 mm.	[19]
Geographical Distribution	Originating from the Central Andes (Peru and Bolivia). It is cultivated from Venezuela to Argentina and Chile and experimentally in New Zealand, Canada, and the United States.	[19]

**Table 3:** Micronutrient composition of *T. tuberosum* (dry weight basis) by ecotype and accession.

Variety/Accession (Ecotype)	Vitamin C (mg/100 g DW)	β-Carotene (µg/g DW)	K (mg/100 g DW)	P (mg/100 g DW)	Ca (mg/100 g DW)	Mg (mg/100 g DW)	Fe (mg/100 g DW)	Zn (mg/100 g DW)	Ref.
Yellow (cultivated)	53–154	18.1–715.95	n.d.	n.d.	n.d.	n.d.	n.d.	n.d.	[4]
Purple (cultivated)	121–446	≈5.65	n.d.	n.d.	n.d.	n.d.	n.d.	n.d.	
Yellow–Purple (cultivated)	90–336	6.91–336.21	n.d.	n.d.	n.d.	n.d.	n.d.	n.d.	
Wild–Purple	213.19	n.d.	1695.04	134.10	34.78	n.d.	7.47	0.424	[23]
Wild–Yellow	220.05	n.d.	1788.87	161.55	35.16	n.d.	7.26	0.275	
Wild–Pink	101.89	n.d.	1797.21	164.15	45.14	n.d.	7.46	0.807	
INIAP-ECU-Izaño (cultivated)	n.d.	n.d.	3250	161	90	n.d.	2.8	n.d.	[10]
White (ECU accession)	n.d.	n.d.	620	240	80	120	n.d.	0.96	[25]
Yellow (ECU accession)	n.d.	n.d.	990	183	100	110	n.d.	0.963	
Purple (ECU accession)	n.d.	n.d.	820	245	25	140	n.d.	1.70	
Milicia Roja (ECU accession)	n.d.	n.d.	660	301	30	140	n.d.	2.713	
Poza Rondador (ECU accession)	n.d.	n.d.	2330	319	650	130	n.d.	0.833	
Green–Yellow (ECU accession)	n.d.	n.d.	1260	336	1430	30	n.d.	0.50	
Various varieties (general range)	n.d.	n.d.	1990	320	6	110	4.2	4.8	[6,7,21]

Note: Values are expressed as mean ± standard deviation. Abbreviations: DW = dry weight; Units—Vit. C: mg/100 g DW; β-carotene: µg/g dry weight; K, P, Ca, Mg, Fe, Zn: mg/100 g dry weight. Abbreviations: ECU = Ecuador (accession origin); DW = dry weight; n.d. = not determined; INIAP = Instituto Nacional de Investigaciones Agropecuarias (Ecuador).

**Table 4:** Total anthocyanin, flavonoid, and phenolic contents (dry weight basis) of *T. tuberosum* according to variety, ecotype, and accession.

Variety/Ecotype/Accession	Total Anthocyanins (C3G, mg/g)	Total Flavonoids (CE, mg/g)	Total Phenolic Compounds (GAE, mg/g)	Reference
Yellow	n.d.	0.047–0.164	1.82–4.05	[4]
Purple	29.20–148.90	0.109–0.453	3.85–11.43	
Yellow–Purple	0.79	0.027–0.086	1.16–2.25	
Fresh *mashua* (raw)	12.44	n.d.	16.41	[35]
Fresh *mashua* (raw)	n.d.	n.d.	8.88	
DP 0224	7.6–9.1	4.8–9.0	14.8–17.1	[36]
ARB 5241	4.4–6.1	6.9–14.2	15.5–22.0	
ARB 5241	n.d.	n.d.	22.2	[18]
var. INIAP-ECU-*izaño*	n.d.	0.04	0.088	[37]
Santo Jonk’ori (1)	n.d.	2.82	2.39	[3]
Achakani *isaño* (2)	n.d.	3.07	2.0	
Ch’iyara *isaño* (3)	n.d.	3.54	10.10	
Tt-02 (yellow)	n.d.	0.16	4.50	[38]
Tt-03 (purple)	n.d.	3.87	8.63	
Tt-11 (yellow)	n.d.	0.21	4.54	
Tt-19 (yellow)	n.d.	0.40	3.87	
Tt-23 (purple)	n.d.	3.98	11.04	
Tt-25 (purple)	n.d.	0.50	8.68	
Kellu (Peru)	0.01	n.d.	7.9	[11]
Chejchi (Peru)	1.03	n.d.	11.9	
Chiar (Bolivia)	3.63	n.d.	22.3	
Kellu (Bolivia)	0.08	n.d.	12.8	
Keni Kellu (Bolivia)	0.11	n.d.	13.1	
Jachir (Bolivia)	0.05	n.d.	9.7	
Asuthi (Bolivia)	0.37	n.d.	8.0	
Yellow *mashua*	n.d.	0.95	2.40	[12]
Pink *mashua*	n.d.	1.40	1.55	
Black *mashua*	n.d.	0.50	3.30	

Note: n.d.: not determined, GAE: gallic acid equivalent; C3G: cyanidin-3-glucoside equivalents, CE: Catechin equivalent.

## Data Availability

No new data were created or analyzed in this study. Data sharing is not applicable to this study.
